# Adsorptive Removal of Direct Azo Dyes from Textile Wastewaters Using Weakly Basic Anion Exchange Resin

**DOI:** 10.3390/ijms24054886

**Published:** 2023-03-03

**Authors:** Monika Wawrzkiewicz, Anna Kucharczyk

**Affiliations:** 1 Department of Inorganic Chemistry, Faculty of Chemistry, Institute of Chemical Sciences, Maria Curie-Sklodowska University in Lublin, M. Curie-Sklodowska Sq. 2, 20-031 Lublin, Poland; 2 MAN Bus Sp.z.o.o., MAN Truck & Bus, Cataphoresis Laboratory, 1 Maj Street 12, 27-200 Starachowice, Poland

**Keywords:** anion exchanger, direct dyes, textile effluents, adsorption, polystyrene resin, direct black 22, direct red 23, direct orange 26

## Abstract

Direct dyes are still widely used for coloring a variety of materials due to their ease of use and the wide range of colors available at a moderate cost of production. In the aquatic environment, some direct dyes, especially the azo type and their biotransformation products, are toxic, carcinogenic and mutagenic. Hence the need for their careful removal from industrial effluents. It was proposed adsorptive retention of C.I. Direct Red 23 (DR23), C.I. Direct Orange 26 (DO26) and C.I. Direct Black 22 (DB22) from effluents using anion exchange resin of tertiary amine functionalities Amberlyst A21 (A21). Applying the Langmuir isotherm model, the monolayer capacities were calculated as 285.6 mg/g for DO26 and 271.1 mg/g for DO23. The Freundlich isotherm model seems to be the better one for the description of DB22 uptake by A21, and the isotherm constant was found to be 0.609 mg^1−1/n^ L^1/n^/g. The kinetic parameters revealed that the pseudo-second-order model could be used for the description of experimental data rather than the pseudo-first-order model or intraparticle diffusion model. The dye adsorption decreased in the presence of anionic and non-ionic surfactants, while their uptake was enhanced in the presence of Na_2_SO_4_ and Na_2_CO_3_. Regeneration of the A21 resin was difficult; a slight increase in its efficiency was observed using 1M HCl, 1 M NaOH and 1 M NaCl solutions in 50% *v*/*v* methanol.

## 1. Introduction

**Figure 1:**
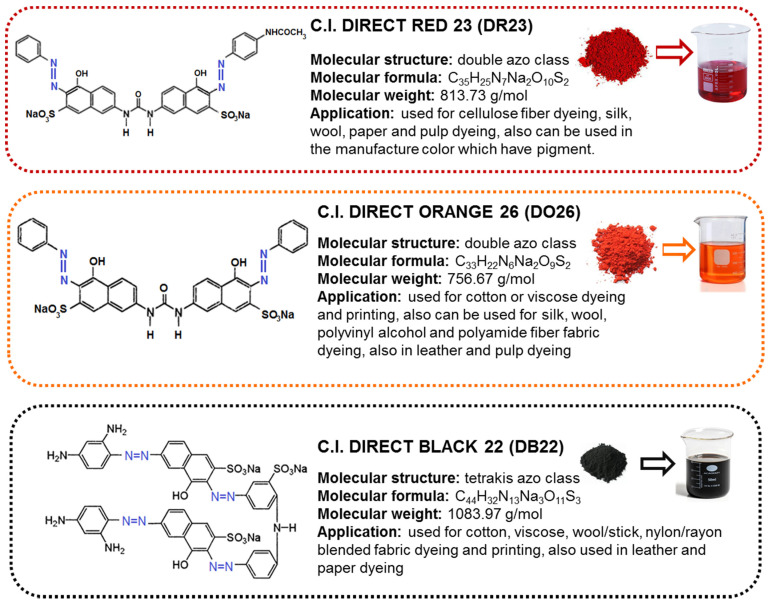
Examples of popular direct dyes–structures and properties.

Direct dyes are a class of dyes that are applied directly to the fiber in an inert or alkaline bath in the presence of sodium chloride (NaCl), carbonate (Na_2_CO_3_) or sulfate (Na_2_SO_4_). They are one of the cheapest groups of dyes and are used for cotton and other cellulosic materials dyeing. They can also be used for the coloring of rayon, silk, wool, paper and leather. Moreover, direct dyes are applied as pH indicators and as biological stains [1]. They are water-soluble salts of sulfonic acids, strong electrolytes almost completely dissociated in dyeing baths into colored anions and sodium cations. More than 75% of all direct dyes are unmetallized azo structures [1]. The great majority of them are disazo or polyazo types with flat structures and high molecular weights, as presented in Figure 1. Wastewater contaminated with azo dyes has become a serious problem due to its negative impact on aquatic ecosystems and humans. Decomposition products of azo dyes are toxic to aquatic organisms, allergenic, mutagenic and carcinogenic to humans and make water unusable. Reduction of azo dyes can be accomplished by human intestinal microflora, skin microflora, environmental microorganisms and by nonbiological means, too [1,2,3]. It is known that high potential health risks are caused by the adsorption of azo dyes and their breakdown products (toxic amines) through the gastrointestinal tract, skin and lungs, as well as the formation of hemoglobin adducts [2,3,4,5].

Effluents from the textile industry vary greatly in composition and differ depending on the dyeing processes used [5,6,7,8]. It is known that 10–25% of textile dyes are lost during dyeing operations, and 2–20% are directly discharged as aqueous effluents [6]. According to Wong et al. [8], approximately 5000–10,000 tons of dyes are released into the wastewater annually. The percentage of unfixed direct dyes that can be disposed of in textile wastewater ranges from 5 to 20% [7]. As presented in Table 1, textile wastewaters are characterized not only by intense coloration but also by high chemical oxygen demand, with a simultaneous low biological oxygen demand, which makes them poorly susceptible to biological treatment processes.

The application of appropriate yet comprehensive methods of removing dyes from wastewater to minimize the above-mentioned parameters is, therefore, extremely challenging. The dye removal methods used most often in practice can be divided into three categories, namely: biological, chemical and physical treatments. The biological dye removal techniques, such as adsorption by microbial biomass, algae or fungal enzyme degradation, used alone are insufficient to completely remove hazardous dyes and auxiliaries. They are usually carried out before discharging wastewater into the environment and have efficiencies of 76–90.1%, with the enzyme degradation method ranking highest on the list [9]. Chemical dye removal methods are considered as costly because of specific equipment and high energy consumption. The above-mentioned disadvantages are compensated by a significant amount of dye removal rate ranging from 88.8 to 99.8% [9]. The average success rate of chemical methods such as advanced oxidation processes, electrochemical destruction, ozonation and ultraviolet irradiation was found to be 97.3%, 88.8%, 94.5% and 99.8%, respectively [9]. Physical dye removal methods are believed to be simple and efficient, with a dye removal yield of 86.8–99%. Adsorption techniques take the lead among other physical methods [8,10] due to ease of operation as well as recovery and reusability of adsorbents, especially of porous structure and high capacity [9,10]. Moreover, its low treatment cost is of great importance. Dotto et al. [11] reported that the adsorption method of wastewater treatment presents a cost of 5.0–200 US $/m^3^, while the other technologies are in the range of 10.0–450 US $/m^3^. A thorough and comprehensive description of adsorption as an effective technique for removing dyes from wastewater, along with key parameters affecting the effectiveness of the technique, can be found in the paper by Rápó and Tonk [12]. Popular trends in the adsorptive removal of dyes from wastewater involve the use of materials such as activated carbons [13], clay and zeolites [14], biosorbents [15], ion exchange resins [16], composites [17] and hybrid adsorbents [18] etc. 

An attempt was made to remove direct dyes such as DB22, DR23 and DO26 using, in particular, biosorbents (i.e., rye straw [19], rice husk [20], wood sawdust [21], orange peel [22]), natural and modified minerals (i.e., halloysite [23], alluvial soil [24] kaolin [25]), or nanocomposites [26,27]. There is little information in the literature on the removal of investigated direct dyes using anion exchange resins [28]. It, therefore, seemed reasonable to undertake research aimed at the use of anion exchange resins, especially of a weak base nature. The aim of this study was to assess the feasibility of using a weakly basic anion exchange resin of polystyrene matrix A21 to remove three direct azo dyes (DB22, DR23 and DO26) from dye baths containing electrolytes and surfactants. The sorption capacity of the resin for the dyes and the kinetic adsorption parameters were determined. In addition, an attempt was made to regenerate the adsorbent.

## 2. Results and Discussion

### 2.1. Isotherm Parameters–Equilibrium Studies

Batch tests carried out at equilibrium made it possible to determine the sorption capacity (q_e_) of the weakly basic anion exchange resin Amberlyst A21 with a polystyrene matrix in relation to the direct dyes such as DB22, DR23 and DO26. Determination of the maximum adsorption is an important part of the research considering the use of a given adsorptive material on an industrial scale as an effective adsorbent capable of removing dyes from wastewater in quantitative amounts. Based on the Langmuir, Freundlich and Temkin adsorption isotherm (Equations (1)–(3)) and the corresponding C_e_/q_e_ vs. C_e_, log(q_e_) vs. log(C_e_) and q_e_ vs. lnC_e_ diagrams, the isotherm parameters were determined [29].

The Langmuir isotherm model suggests that dye absorption occurs on a homogeneous adsorbent surface and results in a monolayer formation in which there are no interactions between adsorbed molecules. The Freundlich and Temkin isotherm models consider the non-homogeneity of the surface and the interactions between adsorbents and dye molecules. One of the assumptions of the Temkin model is that the heat of adsorption of uptaken dye molecules in the layer decreases linearly. 

Analyzing the obtained results (Table 2), it can be concluded that the adsorption of DR23 and DO26 dyes on A21 is most accurately described by the Langmuir adsorption model, as confirmed by the value of the determination coefficient (R^2^) of 0.999. This would suggest the formation of a monolayer of dyes particles on the anion exchanger surface.

**Figure 2:**
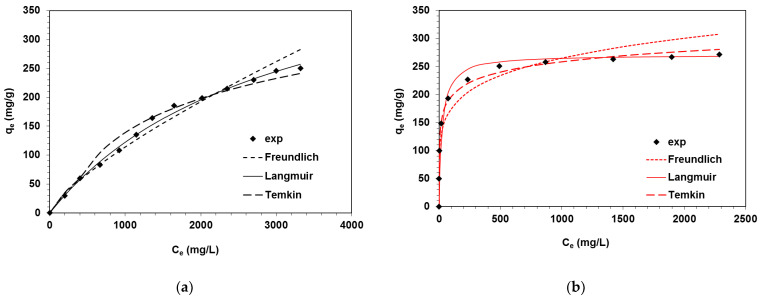

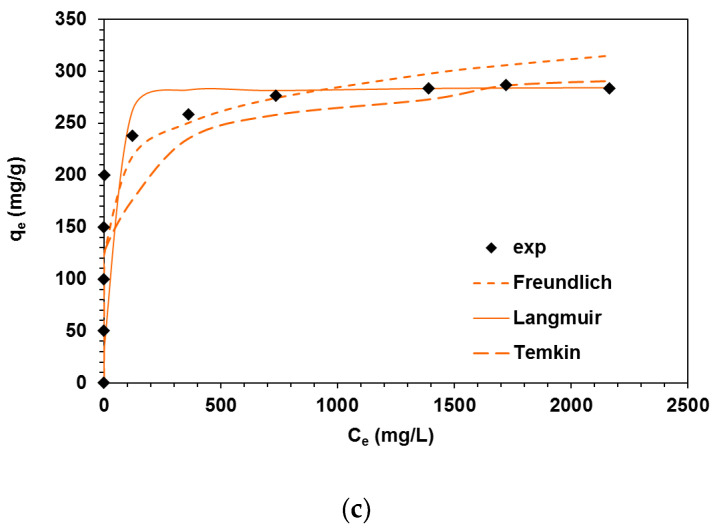
Equilibrium adsorption data with fitting curves corresponding to the Freundlich, Langmuir and Temkin models for (**a**) DB22, (**b**) DR23 and (**c**) DO26 adsorption on A21 resin.

The Langmuir monolayer capacities were calculated as 285.6 mg/g for DO26 and 271.1 mg/g for DR23. The k_L_ constants reflecting the free energy of adsorption were 0.091 L/mg and 0.041 L/mg for DO26 and DR23, respectively. In the DB22-A21 system, the fitting of the Freundlich adsorption model seems to be better for the description of the experimental data, as shown by the determination coefficient (R^2^ = 0.985). Due to the smaller values of the determination coefficients and the shape of the isotherms (Figure 2), the Langmuir and Temkin models proved to be inadequate to describe the adsorption process in the DB22-A21 system.

The maximum adsorption capacity of the weakly basic anion exchange resin A21 for DO26, DR23 and DR22 determined in our studies (Table 2) was compared with the literature values for other adsorbents (Table 3). It can be concluded that the A21 resin has good adsorptive potential to remove direct dyes such as Orange 26, Red 23 and Black 22 from aqueous solutions.

**Figure 3:**
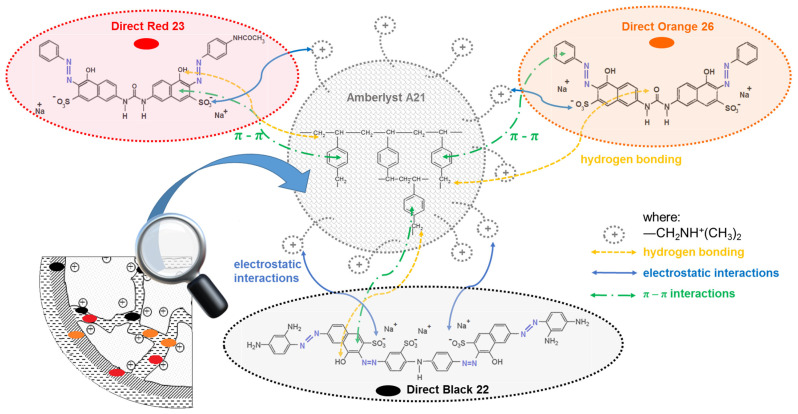
Possible interactions of direct dyes with A-21 resin.

Direct dyes, which are acid dyes as salts of the sulphonic acid (R-SO_3_Na), dissociate into colored anions (RSO_3_^−^) and Na^+^ cations in aqueous baths. Negatively charged dyes anions interact with tertiary amine functional groups (-CH_2_NH^+^(CH_3_)_2_) of the anion exchange resin A21. Not only the electrostatic attraction forces but also π-π interactions may be induced between aromatic benzene rings present in the dyes and resin matrix, as shown in Figure 3. Hydrogen bonding and van der Waals forces can also be considered as a retention mechanism [32,33].

**Figure 4:**
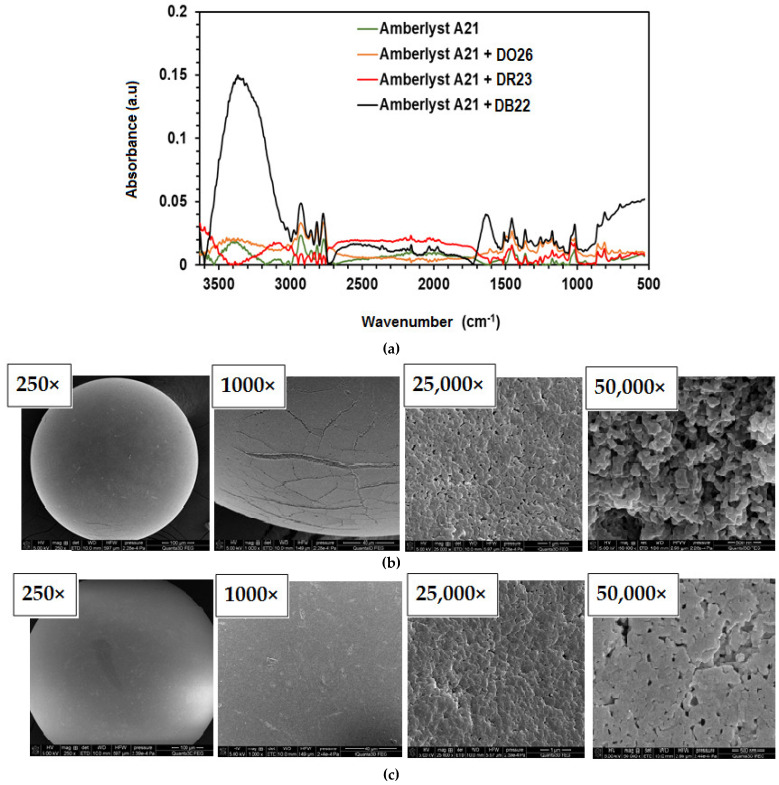
(**a**) ATR-FT-IR spectra of A21 before and after dye sorption and SEM images of the resin (**b**) before and (**c**) after DO26 sorption.

To analyze the adsorption of the direct dyes and to confirm the uptake mechanism, the ATR-FT-IR spectra were recorded before and after sorption, and scanning electron microscopy (SEM) images were obtained, as shown in Figure 4. The intensity changes of the individual bands after the dye sorption on A21 resin were observed in the ATR-FT-IR spectra.

In the ATR-FT-IR spectra of A21 resin before and after sorption of the dyes (Figure 4a), broad bands in the range 3650–3000 cm^−1^ are visible, which correspond to the stretching vibrations of the -OH (presence of water in anion exchanger structure) and =N-H groups. The vibrations of C-H (ν_as_ C-H) and -CH_2_ (ν_as_ -CH_2_) groups in the aromatic rings of the anion exchanger matrix can be found at 3030 cm^−1^ and 2930 cm^−1^ [34]. The C=C stretching vibrations of benzene rings and deformation vibrations of –CH_2_NH^+^(CH_3_)_2_ were assigned at 1620 and 1510 cm^−1^. The stretching and scissoring vibrations of the methylene groups (δ_as_ –CH_2_) of benzene rings were assigned at 1455, 1420 and 1395 cm^−1^. The symmetric and asymmetric vibration of the sulfonic –SO_3_^−^ and –S=O groups at 1085, 1145 and 1013 cm^−1^ after dyes sorption can be noticed, respectively. In the range of 900–750 cm^−1^, there are observed C-H out-of-plane deformation vibrations signals of benzene rings.

Scanning electron microscopy was used to observe the surface morphology of A21 weakly basic resin before and after direct dye uptake (i.e., DO26), as presented in Figure 4b,c. Based on the obtained SEM images, it can be concluded that the studied anion exchanger is in the form of spherical beads with small scratches. There are also visible pores in the structure of A21 which are the sites for dye adsorption. After the adsorption of the dye, a significant reduction in the porosity of the resin and a smoothing of its surface were observed.

### 2.2. Kinetic Parameters–Time Dependence Studies

**Figure 5:**
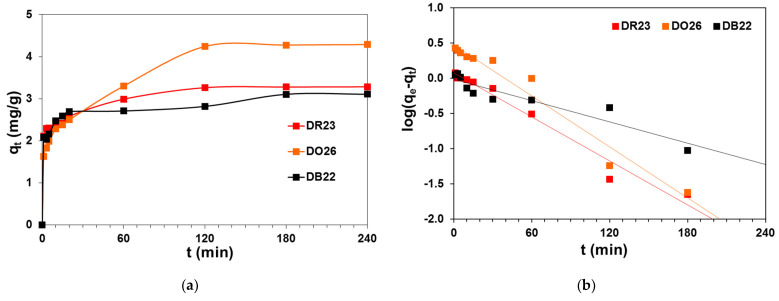

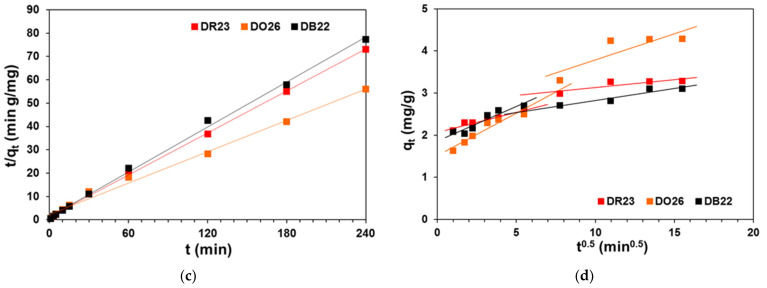
Kinetic plots for (**a**) direct dyes removal on A21 resin as well as (**b**) PFO, (**c**) PSO and (**d**) IPD model considering DB22, DR23 and DO26 dyes adsorption on A21 resin.

In order to design and optimize the adsorption process, it is necessary to determine the amount of adsorbate retained by the adsorbent after a specific time of contact (q_t_) and establish the interactions in the system. The amount of the DR23 and DO26 dyes adsorbed by A21 increased with the increase in the interaction time from 1 to 60 min. It is observed that the time required to obtain significant saturation is 120 min when q_t_ varies insignificantly. For dye solutions of the initial concentration of 100 mg/L, the equilibrium state of the sorption process was established after 180 min, as can be seen in Figure 5a. 

The amounts of adsorbed dye at equilibrium under experimental conditions (q_e,exp_) were 3.2 mg/g for DB22-A21, 3.3 mg/g for DR23-A21 and 4.3 mg/g for DO26-A21 systems, respectively. According to Marin et al. [28], the amount of DR23 adsorbed by IRA402 and XAD7HP resins significantly increased with the increase of time from 15 to 30 min. During the first 30 min, more than half of the amount of DR23 was retained, and the time required to obtain saturation is 90 min [28].

Figure 5b–d illustrates the dye uptake as a function of time. From the slopes and intercepts of the dependences log(q_e_-q_t_) vs. t, t/q_t_ vs. t and q_t_ vs. t^0.5^, the characteristic parameters for the pseudo-first-order Lagergen (PFO), the pseudo-second-order Ho and Mc’Key (PSO) and the Weber and Morris intraparticle diffusion (IPD) equations were calculated, respectively [35,36]. The corresponding kinetic parameters for the selected models are given in Table 4.

The q_e_ values calculated from the PFO model are not equal to q_e,exp_, as evidenced by the values of the determination coefficients R^2^ being in the range 0.883–0.971. Frequently, the pseudo-fist order equation can not be applied for the whole data range of contact time and can be used only for the preliminary stage of adsorption [20]. The data presented deviated from linearity and are probably not consistent with the PFO model. Safa et al. [20] described DO26 adsorption on rice husk, Kuśmierek et al. [23] studied DO26 adsorption on modified halloysite, and Konicki et al. [26] considered the uptake of DO26 by magnetic Fe/graphite core-shall nanocomposite and confirmed that the PFO model could not be used to describe the sorption kinetics of this dye. One can find similar confirmation by analyzing the kinetic parameters calculated for the removal of DR23 on orange peel adsorbent [22] and DB22 on the alluvial soils [24].

The PSO equation can be applied for the description of DB22, DR23 and DO26 adsorption on the A21 resin as the calculated adsorption capacities for this model are much closer to those obtained experimentally compared with the PFO model. The linearity of the t/q_t_ vs. t plot was found (Figure 5c). The k_2_ values were calculated as 0.087, 0.076 and 0.022 g/mg min for DB22, DR23 and DO26, respectively. The R^2^ were 0.998 for DB22, 0.999 for DR23 and 0.993 for DO26. Arami et al. [22], Kuśmierek et al. [21] and Fındık [25] suggested that the removal of the direct dyes such as Orange 26, Red 23 and Black 22 onto biosorbents obeyed the pseudo-second-order kinetic model. 

The rate-limiting step of sorption can be distinguished, taking into consideration diffusion mechanisms such as external diffusion, boundary layer diffusion and intraparticle diffusion. The plot of q_t_ vs. t^0.5^ presented in Figure 5d proved two linear portions. The first part represents the external mass transfer, while the second portion describes intraparticle diffusion. The k_i_ values were calculated as 0.164, 0.094 and 0.202 mg/g min^0.5^ for DR22, DR23 and DO26, respectively. However, the determination coefficients for the IPD model are lower than for the PSO model, indicating that intraparticle diffusion is not the limiting step for the retention of DB22, DR23 and DO26 dyes on the A21 resin.

### 2.3. Solution pH Impact

**Figure 6:**
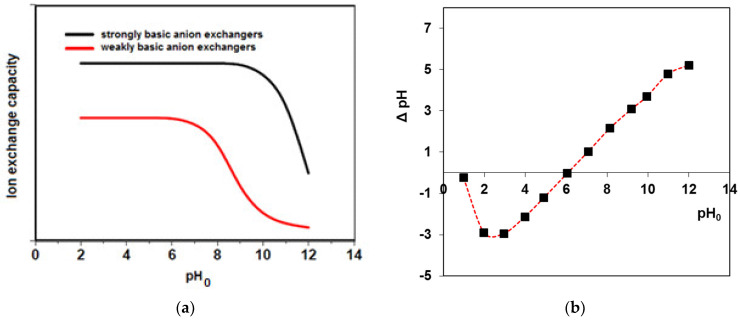
(**a**) Impact of solution pH on the ion exchange capacity of the weakly basic anion exchangers such as A21 resin and (**b**) determination of pH of zero point charge (pH_PZC_) of A21 resin.

Solution pH is a very important parameter affecting the adsorption process as it determines not only the adsorbate charge but also the charge of the adsorbent surface. The most effective working solution pH for the weakly basic anion exchangers is from 2 to 8 [37]. The adsorption capacity of the weak base resin is stable under pH < 8 and does not change with increasing solution pH in the range of 2–8. When the pH of the medium no longer matches the acid dissociation constant (pKa) of the resin functional group, these resins suffer significant capacity loss (Figure 6a). Weak base anion exchangers function poorly above a pH of 9.

The pH_PZC_ of the adsorbent is a very important factor that determines the pH value at which the adsorbent surface has electrical neutrality. The determined pH_PZC_ for A21 resin was found to be 6.01 (Figure 6b). At pH lower than pH_PZC_, A21 is positively charged, while at pH values higher than the pH_PZC_ value, the adsorbent surface is negatively charged. The obtained result is in agreement with one of the proposed mechanisms of dyes retention presented in Figure 3. The negatively charged surface of A21 interacts with the cationic species of the direct dyes.

**Figure 7:**
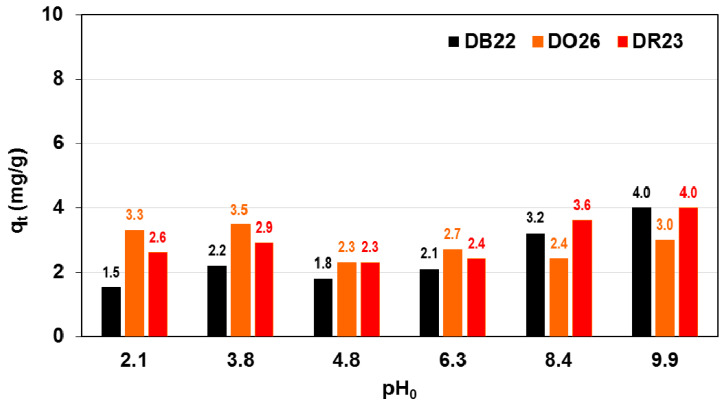
Direct dye adsorption on the A21 weakly basic resin as a function of solution pH.

The effect of pH on the adsorption of DB22, DR23 and DO26 dyes at an initial concentration of 100 mg/L on the A21 resin was investigated in the pH range from 2 to 10 and is shown in Figure 7. 

The amount of the direct dyes adsorbed by the anion exchanger A21 changed slightly with the increasing pH of the solution. The DB22 uptake by A21 increased from 1.5 to 4 mg/g, DO26 retention changed marginally from 3.3 to 3 mg/g, while DR23 adsorption enhancement from 2.6 to 4 mg/g is observed with increasing solution pH. Analysis of the literature data allows the conclusion that the maximum degree of adsorption of DO26 on the rice husk [20], oak sawdust [21] and magnetic Fe/graphite core-shell [26] was observed at pH 3, 2.5 and 4, respectively. DR23 adsorption on the strongly basic anion exchanger Amberlite IRA402 and non-functionalized resin Amberlite XAD7HP reached a maximum value at pH 2 and 7.9 [28]. The optimum medium pH for the uptake of DB22 by the magnetic kaolin-supported zinc ferrite [25] and mushroom waste biosorbent [31] is 7.4.

### 2.4. Electrolytes and Surfactants Effect on Direct Dye Sorption

**Figure 8:**
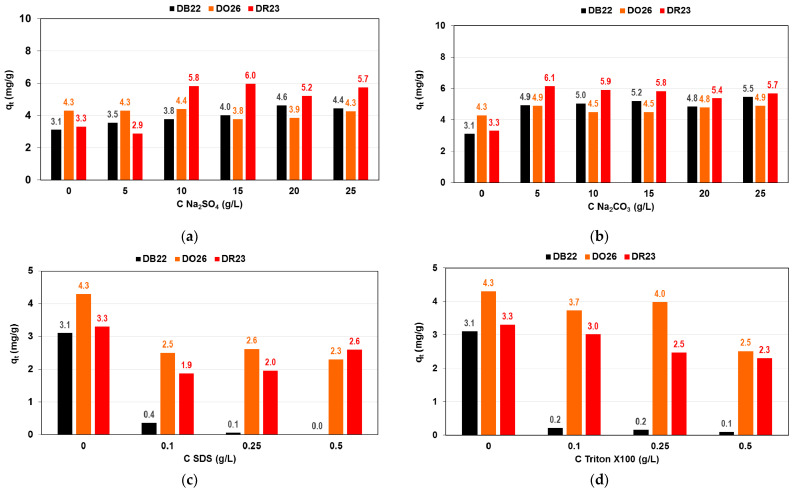
Impact of (**a**) sodium sulfate and (**b**) carbonate as well as (**c**) anionic and (**d**) non-ionic surfactants on DB22, DO26 and DR23 uptake by A21 resin.

Dyeing baths contain significant amounts of electrolytes, which provide suitable conditions for the adsorption of dyes onto the fiber. Together with the surfactants used to remove contaminants from the fiber, they are found in textile effluents. Industrial wastewaters contain various amounts of electrolytes and surfactants depending on the dye type, which considerably affects dye sorption. It was reported by Hessel et al. [7] that the main textile industry loads in Europe relate to salts in the range of 200,000–250,000 tons/year and surfactants at 20,000–25,000 tons/year. The DB22, DR23 and DO26 sorption on A21 resin from the solutions of the initial dye concentration 100 mg/L in the presence of 5–25 g/L Na_2_SO_4_ or Na_2_CO_3_ and anionic (SDS) and non-ionic (Triton X100) surfactants in the amount of 0.1–0.5 g/L was studied (Figure 8).

It was observed that enhancement of the direct dye adsorption by A21 resin with the increasing amount of the electrolytes from 5 to 25 g/L as compared to the adsorption systems without salts (Figure 8a,b). Direct dyes generally tend to aggregate. As the electrolyte concentration in the aqueous solution rise, the aggregation decreases, which favors their adsorption in the resin phase. It was observed that sodium chloride was present in the amount of 0.6–11.6 g/L plays no role in the DO26 adsorption on the halloysites [23]. Safa and Bhatti [20] assessed the effect of MgSO_4_·H_2_O, NaCl, CaCl_2_·2H_2_O, NaNO_3_ and NH_4_NO_3_ (0.01–0.3 M) on the removal of DO26 by rice husk and concluded that salting out effect reduced the solubility of the dye in aqueous phase and increased its uptake by biosorbent.

The sorption of DB22, DR23 and DO26 in the presence of the anionic surfactant SDS and non-ionic Triton X100 was reduced (Figure 8c,d). The amounts of DO26 and DR23 decreased from 4.3 to 2.3 mg/g and from 3.3 to 2.6 mg/g, with an increasing amount of SDS in the system, respectively. The drop in the direct dye uptake is particularly noticeable in the case of DB22. It can be explained by the preferential sorption of the surfactants of a smaller molecular size compared to dyes by the A21 anion exchanger. It was also observed that the capacity of rice husk towards DO26 dropped significantly by adding anionic surfactant SDS [20]. Our previous studies concerning C.I. Direct Yellow 50’s adsorption by the weakly basic anion exchanger also revealed that dye uptake was reduced in the presence of SDS and Triton X100 [38].

### 2.5. Adsorbent Regeneration

An important step in the adsorptive removal of pollutants from wastewater is the ability to regenerate the adsorbent and reuse it. Regeneration of adsorbents can be done by thermal, chemical, microbiological or vacuum methods [39]. Considering that the adsorption of the direct dyes on A21 resin is chemical and physical in nature, different types of eluents were used in the regeneration process. Applying 1 M HCl, 1 M NaOH and 1 M NaCl as regenerant solutions, the percentage of desorption (%D, Equation (7)) of DO26, DR23 and DB22 from the anion exchanger phase was in the range of 1.5–3.3%, 1.9–2.9% and 1.4–3.1%, respectively. 1 M NaOH seems to be the best regenerant among those used (Table 5). The slightly higher degree of desorption of dyes was observed when 1 M HCl, 1 M NaOH and 1 M NaCl solutions were used in the presence of 50% *v*/*v* CH_3_OH, which indicates the breaking of non-specific interactions between the resin matrix and the dyes. 1 M NaOH in 50% *v*/*v* CH_3_OH was able to remove 6.3% of DO26, 5.8% of DR23 and 4.2% of DB22 from the A21 phase. Unfortunately, the results obtained are not satisfactory considering the use of the tested anion exchange resin as a universal adsorbent for removing dyes from wastewater.

## 3. Materials and Methods

### 3.1. Chemicals

Three textile dyes of direct type were used as adsorbates (Figure 1). The dyes were purchased from Boruta–Kolor S.A. (Zgierz, Poland) and used without additional purification. The stock solutions of the individual dye of the initial concentration *C*_0_ = 1000 mg/L were prepared in distilled water, and the working solutions were obtained by dilution.

Amberlyst A21 (DuPont, OH, USA) was used as a dye adsorbent. It is an ion exchange resin in a bead-form, weak base type possessing the tertiary amine functional groups (in the chloride form). The total capacity is 1.3 eq/L. It is a copolymer of styrene and divinylbenzene of macroporous structure. The surface area, total pore volume and average pore diameter were 35 m^2^/g, 0.10 cm^3^/g and 110 Å, respectively, in accordance with the manufacturer’s safety data sheet [40]. The particle diameter is 490–690 µm (uniformity coefficient ≤ 1.8). The water retention capacity is 56–62%. The maximum operating temperature of A21 is 100 °C.

Sodium sulfate, carbonate, chloride, hydroxide, hydrochloric acid and methanol were purchased from Avantor Performance Materials Poland S.A. (Gliwice, Poland). Purified water was obtained using Millipore (UMCS, Lublin, Poland). The surfactants such as anionic sodium dodecyl sulfate and non-ionic 2-[4-(2,4,4-trimethylpentan-2-yl)phenoxy]ethanol Triton X-100 were obtained from Sigma-Aldrich (Taufkirchen, Germany).

### 3.2. Experimental Methods–Adsorption and Desorption

The adsorption parameters were determined using a static method. For this purpose, 0.5 g of A21 resin was weighted in the conical flasks, and then 50 mL of the dye solutions of specified concentrations were added. The flasks were placed in a mechanical shaker Elpin 358+ (Lubawa, Poland) of 180 cycles per minute (amplitude 8) at room temperature, and the agitation was carried out at 1, 3, 5, 10, 15, 30, 60, 120, 180 and 240 min (kinetic studies), 24 h (adsorption isotherms), 15 min (effects of electrolytes and surfactants as well as pH on dye adsorption efficiency). The suspensions were filtered, and the contents of the DO26, DR23 and DB22 were determined spectrophotometrically using Cary 60 Agilent (Santa Clara, CA, USA) apparatus at 494 nm, 502 nm and 504 nm, respectively. The adsorption experiments were performed in triplicate with reproducibility ±5%.

To determine the adsorption isotherms of the dyes on A21, solutions were prepared of increasing initial concentrations of dyes ranging from 500 to 5000 mg/L. The obtained equilibrium adsorption data were fitted to the popular isotherm models such as the Freundlich (Equation (1)), Langmuir (Equation (2)) and Temkin (Equation (3)) using linear forms of the equations [29]:

(1) $$\frac{C_{e}}{q_{e}}=\frac{1}{Q_{0}k_{L}}+\frac{C_{e}}{Q_{0}}$$

(2) $$\log\;q_{e}=\log\;k_{F}+\frac{1}{n}\log\;C_{e}$$

(3) $$q_{e}=\left(\frac{RT}{b_{T}}\right)\ln A+\left(\frac{RT}{b_{T}}\right)\ln C_{e}$$

where: *C_e_*—equilibrium concentration of direct dye solutions (mg/L), *Q*_0_—the monolayer adsorption capacity (mg/g), *k_L_*—the Langmuir constant (relating to the free energy of adsorption) (L/mg), *k_F_* (mg^1−1/n^ L^1/n^/g) and 1/n—the Freundlich constants concerning adsorption capacity and the surface heterogeneity, respectively, *R*—gas constant (8.314 J/mol K), *T*—temperature (K), *A* (L/g) and *b_T_* (J/mol)—the Temkin constants and the corresponding *C_e_/q_e_* vs. *C_e_*, log(*q_e_*) vs. log(*C_e_*) and *q_e_* vs. ln*C_e_* diagrams, the isotherm parameters were determined.

The attenuated total reflectance Fourier transform infrared spectroscopy (ATR-FT-IR) technique was used to describe the interaction of the dyes with A21 resin (apparatus Cary 630, Agilent, Santa Clara, CA, USA). Additionally, the surface morphology of the A21 anion exchanger before and after dye sorption was determined at 250×, 1000×, 25,000× and 50,000× magnification using scanning electron microscopy (Quanta 3D, FEG, Hillsboro, OR, USA).

The DO26, DR23 and DB22 solutions of 100 mg/L initial concentration were used to describe the sorption kinetics. To describe the sorption kinetics of the individual dyes on the A21 resin, the pseudo-first-order Lagergren equation (PFO) (Equation (4)), the pseudo-second-order Ho and Mc’Key equation (PSO) (Equation (5)) and the Weber and Morris intraparticle diffusion (IPD) model (Equation (6)) were used [35,36]:

(4) $$\log(q_{e}-q_{t})=\log\left(q_{t}\right)-\frac{k_{1}}{2.303}t$$

(5) $$\frac{t}{q_{t}}=\frac{1}{k_{2}q_{e}^{2}}+\frac{1}{q_{e}}t_{\;}$$

(6) $$q_{t}=k_{i}t^{0.5}$$

where: *q_e_*—adsorption capacity (mg/g), *q_t_* (mg/g)—amount of direct dye adsorbed at time *t* (min), *k*_1_ (1/min), *k*_2_ (g/mg min) and *k_i_* (mg/g min^0.5^)—rate constants of sorption calculated from the PFO, PSO and IPD equations, respectively.

In order to determine the effect of auxiliaries on the sorption efficiency of the dyes, solutions containing 100 mg/L of DO26, DR23 or DB22 in the presence of electrolytes and surfactants were prepared of the following compositions: 5, 10, 15, 20 and 25 g/L sodium sulfate or sodium carbonate; 0.1, 0.25, 0.5 mg/L Triton X-100 or SDS. 

The dye solution of 100 mg/L concentration and increasing initial pH from 2.9 to 9.9 were prepared to investigate the effect of pH on the dye uptake by A21 after 240 min of equilibration. 1 M NaOH and 1 M HCl were used to obtain the specified pH of the solutions using pH-metre CP-411 (Elmetron, Zabrze, Poland). The pH of zero point charge (pH_PZC_) of the A21 anion exchanger was determined according to the solid addition method [41], too. Amberlyst A21 anion exchanger in the amount of 0.5 g was immersed in 50 mL of 0.01 M KNO_3_ solutions in which the initial pH values from 2 to 9 were adjusted using 1 M HCl or 1 M NaOH and left for 24 h. The final pH of the solutions was measured after 24 h. The pH_PZC_ value was determined based on the curve pH_0_ versus ΔpH (difference between the initial pH (pH_0_) and the final pH–measure after 24 h (pH_f_), ΔpH = pH_0_–pH_f_). After the sorption process (50 mL solution of 100 mg/L DO26, DR23 or DB22, 0.5 g of A21, t = 240 min., 180 cycles per minute, amplitude 8, room temperature), an attempt was made to regenerate the resin by applying the following experimental conditions: 0.5 g of A21 with adsorbed dyes (3.3 mg/g DR23; 4.3 mg/g DO26; 3.1 mg/g DB22) was placed in a conical flask and shaken with 50 mL of previously prepared eluents (1 M NaOH, 1 M HCl, 1 M NaCl, 50% *v*/*v* CH_3_OH, 1 M NaOH + 50% *v*/*v* CH_3_OH, 1 M HCl + 50% *v*/*v* CH_3_OH, 1 M NaCl + 50% *v*/*v* CH_3_OH). The desorption was carried out at 180 cpm (amplitude 8) for 180 min. After filtration of the solutions, the dye contents were determined spectrophotometrically and expressed as the percentage of desorption (%D):

(7) $$\%D=\frac{m_{des}}{m_{ads}}100\%$$

where: *m_des_*—mass of desorbed dye (mg), *m_ads_*—mass of adsorbed dye (mg).

## 4. Conclusions

The study evaluated the applicability of the weakly basic anion exchanger of polystyrene matrix Amberlyst A21 for C.I. Direct Black 22, C.I. Direct Red 23 and C.I. Direct Orange 26 dyes removal from wastewater. Experimental conditions such as the dye’s initial concentration, time of phase contact and auxiliaries addition (Na_2_SO_4_, Na_2_CO_3_, SDS and TX-100) were taken as parameters determining the adsorption efficiency of A21 resin. The Langmuir isotherm model (R^2^ = 0.999) described the adsorption of DR23 and DR26 at equilibrium with the largest capacities of 271.1 mg/g and 285.6 mg/g, respectively. The Freundlich isotherm model with k_F_ = 0.609 mg^1−1/n^L^1/n^/g seems to be the better one for interpretation of DR22 uptake by the anion exchanger, as R^2^ was equal to 0.985. The pseudo-second-order kinetic model is adequate for the characterization of dyes-resin adsorption systems, as evidenced by the values of the determination coefficients R^2^ being in the range of 0.993–0.999. The PSO rate constants k_2_ were calculated to be 0.087, 0.076 and 0.022 g/mg min for DB22, DR23 and DO26, respectively. The dyes’ adsorption was influenced by the electrolytes (Na_2_SO_4_ and Na_2_CO_3_) and surfactant (SDS and Triton X100) presence in the dyeing baths. Salts present in dye solutions increased their adsorption, while surfactants decreased their uptake. Interactions between adsorbed dyes and the resin are so strong that the process of their desorption using not only aqueous solutions of 1M HCl, 1 M NaOH and 1 M NaCl but also in 50% *v*/*v* methanol is not satisfactory. The best results were obtained using 1 M NaOH in 50% *v*/*v* CH_3_OH: 6.3% of DO26, 5.8% of DR23 and 4.2% of DB22 were desorbed from the A21 phase. However, the described results of the study have great cognitive significance in assessing the suitability of adsorbent materials as effective for removing dyes from wastewater and dyeing baths in the textile industry.

## Figures and Tables

**Table 1:** Parameters of textile wastewater [1].

Parameter	Value
pH	2–14
Color (Pt-Co) BOD_5_ (mg/L) COD (mg/L) TDS (mg/L) TSS (mg/L) Turbidity (NTU)	>200 110–5500 131–17,900 47.9–6106 64–23,900 15–5700

Where: BOD_5_—biological oxygen demand, COD—chemical oxygen demand, TDS—total dissolved solids, TSS—total suspended solids, NTU—Nephelometric Turbidity Units.

**Table 2:** Isotherm parameters determined from the Langmuir, Freundlich and Temkin models calculated for the adsorption of DB22, DR23 and DO26 on A21 resin at room temperature using linear regression.

Parameters	DB22	DR23	DO26
Q_0_ (mg/g) k_L_ (L/mg) R^2^	491.5 0.0003 0.970	271.1 0.041 0.999	285.6 0.091 0.999
K_F_ (mg^1−1/n^ L^1/n^/g) 1/n R^2^	0.609	76.2	117.1
	0.757	0.18	0.129
	0.985	0.900	0.729
A (L/g) b_T_ (J/mol) R^2^	5.1	16.5	632.1
	29.1 0.962	93.2 0.984	118.5 0.886

**Table 3:** Screening of adsorbents applied for DO26, DR23 and DB22 removal using different types of adsorbents.

Sorbent	Direct Dye	Isotherm Model Used and/or Description of Equilibrium Results	Ref.
Mechanically and chemically modified rye straw	Direct Orange 26	Freundlich: k_F_ = 0.941 mg^1−1/n^ L^1/n^/g, pH = 5–6, a.d. = 5 g/200 mL	[19]
Rice husk		Langmuir: Q_0_ = 66.67 mg/g pH = 3, a.d. = 0.06 g/50 mL	[20]
Untreated halloysites (H-NM), halloysites modified by sodium benzoate (H-SB) and sulfuric acid (H-SA1 and H-SA2)		Langmuir: Q_0_ = 49.13 mg/g for H-NM, Q_0_ = 56.04 mg/g for H-SB, Q_0_ = 229.3 mg/g for H-SA1, Q_0_ = 49.13 mg/g for H-SA2 pH = 2.5, a.d. = 0.02 g/20 mL	[23]
Cherry wood sawdust (CWS) Pine wood sawdust (PWS) Oak wood sawdust (OWS)		Langmuir: Q_0_ = 3.48 mg/g for CWS, Q_0_ = 3.49 mg/g for PWS, Q_0_ = 3.73 mg/g for OWS, pH = 2.5–7, a.d. = 0.02 g/20 mL	[21]
magnetic Fe-graphite core-shell nanocomposite		Langmuir: Q_0_ = 28.3 mg/g pH = 2.5–7, a.d. = 0.03 mg/150 mL	[26]
Amberlyst A21 (weakly basic anion exchange resin in Cl^−^)		Langmuir: Q_0_ = 285.6 mg/g, pH = 8.4, a.d. = 0.03 mg/150 mL	This study
Orange peel adsorbent	Direct Red 23	Langmuir: Q_0_ = 21.05 mg/g pH = 2, a.d. = 2 g/250 mL	[22]
Amberlite IRA402 (strongly basic anion exchange resin in Cl^−^); Amberlyst XAD7HP (non-functionalized acrylic resin)		Langmuir: Q_0_ = 21 mg/g pH = 2, a.d. = 0.5 g/50 mL for IRA402 Q_0_ = 27 mg/g, pH = 7.9, a.d.= 0.5 g/50 mL for XAD7HP	[28]
Biocomposite (metal-organic framework) modified with different amounts of 3-aminopropyl-trimethoxysilane for preparing different MILs-53A/NH_2_ adsorbents; Chitosan; MIL-53A/NH_2_–chitosan bionanocomposit (SFMOF/BM)		Langmuir: Q_0_ = 3333–10,000 mg/g, pH = 2.1, a.d. = 2 g/100 mL for different MILs-53A/NH_2_ Q_0_ = 3360 mg/g, pH = 2.1, a.d. = 2 g/100 mL for Chitosan Q_0_ = 12,500 mg/g, pH = 2.1, a.d. = 2 g/100 mL for SFMOF/BM	[27]
Amberlyst A21 (weakly basic anion exchange resin in Cl^-^)		Langmuir: Q_0_ = 271.1 mg/g, pH = 8,4, a.d. = 0.5 mg/50 mL	This study
Alluvial soil	Direct Black 22	Freundlich model: k_F_ = 8.6 kg^−1^ mg^1−1/n^L^1/n^, a.d. = 5 g/50 mL	[24]
Polyacrylonitrile membranes incorporated with graphene oxide functionalized with safranin dye (hPAN@GO-SF)		Adsorption capacity: 148–205 mg/g in the saline and single system	[30]
Synthesized magnetic kaolin-supported zinc ferrite		Langmuir: Q_0_ = 8.404 mg/g, pH = 7.4, a.d. = 1 g/200 mL	[25]
Spent mushroom waste		Langmuir: Q_0_ = 15.46 mg/g, pH = 7.4, a.d. = 1 g/200 mL	[31]
Amberlyst A21 (weakly basic anion exchange resin in Cl^-^)		Freundlich model: k_F_ = 0.609 mg^1−1/n^L^1/n^/g, pH = 8,4, a.d. = 5 g/50 mL	This study

Where: a.d.—adsorbent dose.

**Table 4:** Kinetic parameters calculated from PFO, PSO and IPD models for direct dye sorption on A21 resin in aqueous solutions of 100 mg/L initial concentration.

Dye	q_e,exp_ (mg/g)	PFO			PSO			IPD	
		q_e_ (mg/g)	k_1_ (1/min)	R^2^	q_e_ (mg/g)	k_2_ (g/mg min)	R^2^	k_i_ (mg/g min^0.5^)	R^2^
DB22	3.2	0.96	0.012	0.883	3.1	0.087	0.998	0.164	0.866
DR23	3.3	1.2	0.024	0.971	3.3	0.076	0.999	0.094	0.914
DO26	4.3	3.0	0.028	0.965	4.5	0.022	0.993	0.202	0.893

**Table 5:** Dyes desorption results.

Eluent	%D		
	DO26	DR23	DB22
1 M HCl	1.5	2.7	2.1
1 M NaOH	3.3	2.9	3.1
1 M NaCl	2.1	1.9	1.4
50% *v*/*v* CH_3_OH	1.2	1.7	1.9
1 M HCl–50% *v*/*v* CH_3_OH	4.3	3.3	3.1
1 M NaOH–50% *v*/*v* CH_3_OH	6.3	5.8	4.2
1 M NaCl–50% *v*/*v* CH_3_OH	1.7	2.2	1.9

## Data Availability

The data presented in this study are available in this article.

## References

[1] Gürses A., Açıkyıldız M., Güneş K., Gürses M.S. (2016). Dyes and Pigments.

[2] Chung K.-T. (2016). Azo dyes and human health: A review. J. Environ. Sci. Health C.

[3] Puvaneswari N., Muthukrishnan J., Gunasekaran P. (2006). Toxicity assessment and microbial degradation of azo dyes. Indian J. Exp. Biol..

[4] Hashemi S.H., Kaykhaii M., Dalu T., Tavengwa N. (2022). Chapter 15—Azo Dyes: Sources, Occurrence, Toxicity, Sampling, Analysis, and Their Removal Methods.

[5] Lellis B., Fávaro-Polonio C.Z., Pamphile J.A., Polonio J.C. (2019). Effects of textile dyes on health and the environment and bioremediation potential of living organisms. Biotechnol. Res. Innov..

[6] Gita S., Shukla S.P., Deshmukhe G., Choudhury T.G., Saharan N., Singh A.K. (2021). Toxicity evaluation of six textile dyes on growth, metabolism and elemental composition (C, H, N, S) of *Microalgae Spirulina* platensis: The environmental consequences. Bull. Environ. Contam. Toxicol..

[7] Hessel C., Allegre C., Maisseu M., Charbit F., Moulin P. (2007). Guidelines and legislation for dye house effluents. J. Environ. Manag..

[8] Wong S., Ghafar N.A., Ngadi N., Razmi F.A., Inuwa I.M., Mat R., Amin N.A.S. (2020). Effective removal of anionic textile dyes using adsorbent synthesized from coffee waste. Sci. Rep..

[9] Katheresan V., Kansedo J., Lau S.Y. (2018). Efficiency of various recent wastewater dye removal methods: A review. J. Environ. Chem. Eng..

[10] Nguyen C.T., Sabarathinam S., Sridharan S., Al Salhi M.S., Devanesan S., Shanmuganathan R., Lan Chi N.T. (2022). Comparison of Simarouba glauca seed shell carbons for enhanced direct red 12B dye adsorption: Adsorption isotherm and kinetic studies. Food Chem. Toxicol..

[11] Dotto G.L., Santos J.M.N., Rodrigues I.L., Rosa R., Pavan F.A., Lima E.C. (2015). Adsorption of Methylene Blue by ultrasonic surface modified chitin. J. Colloid Interface Sci..

[12] Rápó E., Tonk S. (2021). Factors affecting synthetic dye adsorption; desorption studies: A review of results from the last five years (2017–2021). Molecules.

[13] Raji Y., Nadi A., Rouway M., Jamoudi Sbai S., Yassine W., Elmahbouby A., Cherkaoui O., Zyade S. (2022). Efficient Adsorption of Methyl Orange on nanoporous carbon from agricultural wastes: Characterization, kinetics, thermodynamics, regeneration and adsorption mechanism. J. Compos. Sci..

[14] Imessaoudene A., Cheikh S., Hadadi A., Hamri N., Bollinger J.-C., Amrane A., Tahraoui H., Manseri A., Mouni L. (2023). Adsorption performance of zeolite for the removal of Congo Red Dye: Factorial design experiments, kinetic, and equilibrium studies. Separations.

[15] Jóźwiak T., Filipkowska U., Bakuła T., Bralewska-Piotrowicz B., Karczmarczyk K., Gierszewska M., Olewnik-Kruszkowska E., Szyryńska N., Lewczuk B. (2023). The use of chitin from the Molts of Mealworm (*Tenebrio molitor*) for the removal of anionic and cationic dyes from aqueous solutions. Materials.

[16] Naim M.M., Al-harby N.F., El Batouti M., Elewa M.M. (2022). Macro-reticular ion exchange resins for recovery of direct dyes from spent dyeing and soaping liquors. Molecules.

[17] Zghal S., Jedidi I., Cretin M., Cerneaux S., Abdelmouleh M. (2023). Adsorptive removal of Rhodamine B dye using carbon graphite/cnt composites as adsorbents: Kinetics, isotherms and thermodynamic study. Materials.

[18] Ezzat A.O., Tawfeek A.M., Rajabathar J.R., Al-Lohedan H.A. (2022). Synthesis of new hybrid structured magnetite crosslinked poly ionic liquid for efficient removal of Coomassie Brilliant Blue R-250 dye in aqueous medium. Molecules.

[19] Tomczak E., Tosik P. (2014). Sorption equilibrium of azo dyes Direct Orange 26 and Reactive Blue 81 onto a cheap plant sorbent. Ecol. Chem. Eng. S..

[20] Safa Y., Bhatti H.N. (2011). Kinetic and thermodynamic modeling for the removal of Direct Red-31 and Direct Orange-26 dyes from aqueous solutions by rice husk. Desalination.

[21] Kuśmierek K., Gałan M., Kamiński W., Świątkowski A. (2020). Zastosowanie trocin jako tanich sorbentów do usuwania barwników azowych z wody. Przem. Chem..

[22] Arami M., Limaee N.Y., Mahmoodi N.M., Tabrizi N.S. (2005). Removal of dyes from colored textile wastewater by orange peel adsorbent: Equilibrium and kinetic studies. J. Colloid Interf. Sci..

[23] Kuśmierek K., Światkowski A., Wierzbicka E., Legocka I. (2020). Enhanced adsorption of Direct Orange 26 dye in aqueous solutions by modified halloysite. Physicochem. Probl. Miner. Process..

[24] da Silva Alexandre J.I., Martins dos Santos Neto S., Paiva Coutinho A., dos Anjos Tenório de Melo T., Amaral Pastich Gonçalve E., Salgueiro Gondim M.V., Dantas Antonino A.C., Carvalho de Gusmão da Cunha Rabelo A.E., de Oliveira A.L. (2020). Sorption of the Direct Black 22 dye in alluvial soil. Rev. Ambient. Água.

[25] Fındık S. (2022). Removal of diazo dye Direct Red 28 and tetra azo dye Direct Black 22 using synthesized magnetic kaolin supported zinc ferrite. Acta Chim. Slov..

[26] Konicki W., Hełminiak A., Arabczyk W., Mijowska E. (2017). Removal of anionic dyes using magnetic Fe@graphite core-shell nanocomposite as an adsorbent from aqueous solutions. J. Colloid Interf. Sci..

[27] Allahbakhshi M., Mahmoodi N.M., Mosaferi M., Kazemian H., Aslani H. (2022). Synthesis of functionalized metal-organic framework metal-organic framework (MIL-53)/Chitosan for removing dye and pharmaceutical. Surf. Interfaces.

[28] Marin N.M., Ficai A., Constantin L.A., Motelica L., Trusca R. (2022). New chelate resins prepared with Direct Red 23 for Cd^2+^, Ni^2+^, Cu^2+^ and Pb^2+^ removal. Polymers.

[29] Foo K.Y., Hameed B.H. (2010). Insights into the modeling of adsorption isotherm systems. Chem. Eng. J..

[30] Neves T.D.F., Camparotto N.G., Brião G.D.V., Mastelaro V.R., Renato Falcãoa D.R., Vieira M.G.A., Prediger P. (2022). Graphene oxide-safranin modified@polyacrylonitrile membranes for water purification: Reuse and mechanism based on theoretical calculations and XPS analysis. J. Water Process Eng..

[31] Alhujaily A., Yu H., Zhang X., Ma F. (2020). Adsorptive removal of anionic dyes from aqueous solutions using spent mushroom waste. Appl. Water Sci..

[32] Wawrzkiewicz M., Podkościelna B. (2022). Innovative Polymer Microspheres with Chloride Groups Synthesis, Characterization and Application for Dye Removal. Processes.

[33] Podkościelna B., Wawrzkiewicz M., Klapiszewski Ł. (2021). Synthesis, Characterization and Sorption Ability of Epoxy Resin-Based Sorbents with Amine Groups. Polymers.

[34] Bartholin M. (1981). Styrene-divinylbenzene copolymers, 3 revisited IR analysis. Makromol. Chem..

[35] Ho Y.S., McKay G. (1998). Kinetics models for the sorption of dye from aqueous solution by wood. Process Saf. Environ. Prot..

[36] Weber W., Morris J. (1963). Kinetics of adsorption on carbon from solutions. J. Sanit. Eng. Div..

[37] Harland C.E. (1994). Ion Exchange: Theory and Practice.

[38] Wawrzkiewicz M., Polska-Adach E. (2021). Physicochemical interactions in systems C.I. Direct Yellow 50—Weakly basic resins: Kinetic, equilibrium, and auxiliaries addition aspects. Water.

[39] Lee S., Lee J.-S., Song M.-K., Ryu J.-C., An B., Lee C.-G., Park C., Lee S.-H., Choi J.-W. (2015). Effective regeneration of an adsorbent for the removal of organic contaminants developed based on UV radiation and toxicity evaluation. React. Funct. Polym..

[40] https://www.lenntech.com/Data-sheets/Dupont-Amberlyst-A21-L.pdf.

[41] Wan Ngah W.S., Hanafiah M.A.K.M. (2008). Adsorption of copper on rubber (Hevea brasiliensis) leaf powder: Kinetic, equilibrium and thermodynamic studies. Biochem. Eng. J..

